# Imaging of Cerebral Venous Thrombosis

**DOI:** 10.3390/life12081215

**Published:** 2022-08-10

**Authors:** Jean-Claude Sadik, Dragos Catalin Jianu, Raphaël Sadik, Yvonne Purcell, Natalia Novaes, Edouard Saragoussi, Michaël Obadia, Augustin Lecler, Julien Savatovsky

**Affiliations:** 1 Department of Diagnostic Neuroradiology, Hôpital Fondation A. de Rothschild, 75019 Paris, France; 2 First Department of Neurology, “Victor Babes” University of Medecine and Pharmacy, 300041 Timisoara, Romania; 3 Geriatrics Rehabilitation, Hospital Riviera Chablais—la Providence, 1800 Vevey, Switzerland; 4 Department of Neurology, Stroke Unit, Hôpital Fondation A. de Rothschild, 75019 Paris, France

**Keywords:** cerebral vein, cerebral venous thrombosis, venous stroke, dural sinus thrombosis, intracranial hypertension, MR venography, MRI, CT venography

## Abstract

Cerebral venous thrombosis is a rare cause of stroke. Imaging is essential for diagnosis. Although digital subtraction angiography is still considered by many to be the gold standard, it no longer plays a significant role in the diagnosis of cerebral venous thrombosis. MRI, which allows for imaging the parenchyma, vessels and clots, and CT are the reference techniques. CT is useful in case of contraindication to MRI. After presenting the radio-anatomy for MRI, we present the different MRI and CT acquisitions, their pitfalls and their limitations in the diagnosis of cerebral venous thrombosis.

## 1. Introduction

Cerebral venous thrombosis is a rare but potentially serious disease. It accounts for 0.5% to 1% of all strokes in adult populations. It can occur at any age but more frequently affects young people, particularly pregnant women and women on estrogen–progestin contraceptives.

Cerebral imaging is fundamental for detecting cerebral venous thrombosis and the parenchymal complications. Cerebral venous thrombosis is not diagnosed without imaging. Noninvasive cerebral imaging (CT and MRI) has completely replaced digital subtraction angiography (DSA), which is now used to guide exceptional endovascular treatments of the most severe forms that worsen despite effective anticoagulation.

Imaging cerebral venous thrombosis has a poor reputation because in the absence of good knowledge of the anatomy and the different results of CT and MRI, it is exposed to pitfalls that can be easily avoided [1].

This review are to illustrate the anatomy of the cerebral venous system and the accuracy of CT and MRI for cerebral venous thrombosis diagnosis. We present the main findings and pitfalls of these two imaging modes.

## 2. Pathophysiology

Cerebral venous thrombosis is secondary to a local or general imbalance of the coagulation system that leads to a thrombus developing in the dural sinuses or cerebral veins. Thrombosis of the dural sinuses leads to a delay in venous emptying and a decrease in the reabsorption of cerebral spinal fluid. Because of the obstruction, there is an increase in venous and capillary pressure.

If the collaterals are of good quality, the only symptoms are those related to intracranial hypertension. However, if the collaterals are insufficient or with extension to the cortical veins, the intracranial pressure may increase until it exceeds the cerebral perfusion pressure, thus increasing the ischemic risk.

Cerebral venous thrombosis leads to the formation of areas of focal edema related to increased retrograde pressure, characterized by venous dilatation, petechiae that are sometimes confluent and lead to hematomas, and focal non-systematized ischemic damage. Parenchymal lesions occur in 60% of patients with cerebral venous thrombosis and differ significantly from those in arterial stroke because they cross arterial boundaries, have a hemorrhagic component in nearly two-thirds of cases, and often feature a combination of vasogenic and cytotoxic edema.

## 3. Clinical Presentations

A wide range of symptoms should cause suspicion of cerebral venous thrombosis. These symptoms, which progressively worsen and fluctuate, are mostly nonspecific. The condition is diagnosed by imaging at 7 days on average after the onset of clinical symptoms.

**Figure 1:**
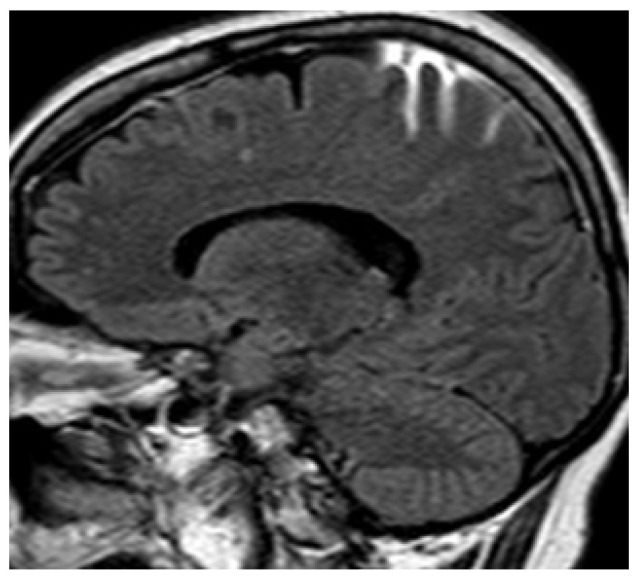
Sagittal fluid attenuated inversion recovery (FLAIR) image. Spontaneous subarachnoid hemorrhage in the precentral, central, and postcentral sulci in the context of cerebral venous thrombosis. Some cortical veins are visible, but their thrombosis cannot be verified because they are drowned in the spontaneous FLAIR signal of the subarachnoid hemorrhage.

The most common symptoms are headache that indicates intracranial hypertension, diplopia related to a sixth nerve palsy, papilledema [2], and an altered mental status. If symptoms appear suddenly, they can also reveal a subarachnoid hemorrhage [3,4,5] without arterial cause [Figure 1].

Focal neurological motor or sensory deficits, aphasia, or hemianopsia are most often the result of a hematoma or parenchymal ischemia easily diagnosed on MRI and CT. Focal or generalized seizures are more common in cerebral venous thrombosis than in other causes of stroke.

Cerebral venous thrombosis occurs most often in populations with predisposing risk factors such as pro-thrombotic factors (genetic or acquired), autoimmune diseases, use of hormones for contraception or hormone replacement therapy, pregnancy, being in the postpartum period, heart failure, brain tumors (especially hematological diseases), and infections; however, in about 30% of cases, no cause is found.

## 4. Cerebral Venous Anatomy

The clinical presentation depends on the location and extension of the thrombus as well as parenchymal abnormalities [2]. Cerebral veins do not have valves [6] and are largely anastomosed to each other, which allows for the development of collateral circulation in case of occlusion.

Anatomical description is difficult due to the great variability of the cortical veins. However, the large deep veins and the dural sinuses can be easily identified. The walls of dural sinuses are formed by the dural mater.

**Figure 2:**
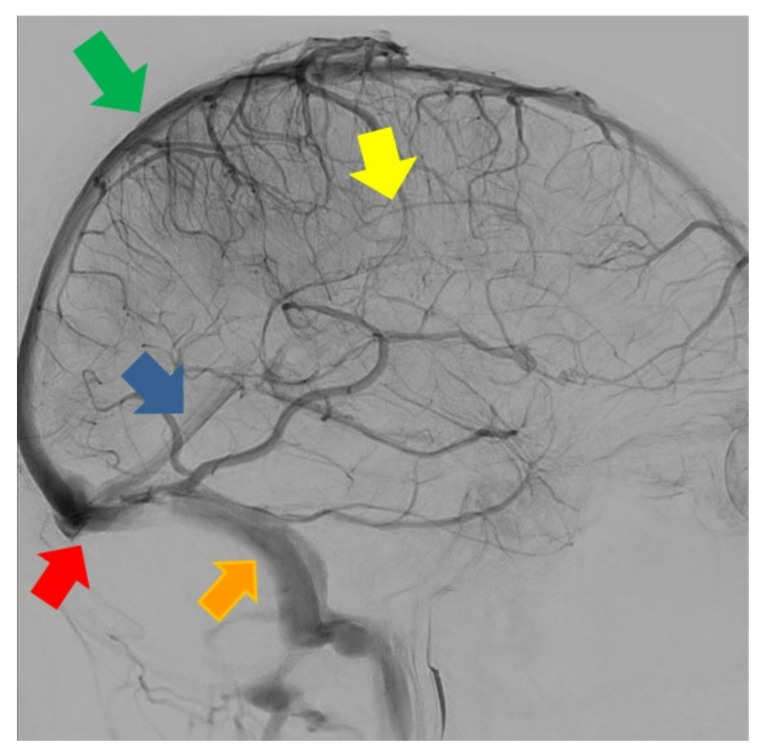
Digital subtraction angiography, sagittal plane. Green arrow: superior sagittal sinus. Yellow arrow: inferior sagittal sinus. Blue arrow: straight sinus. Orange arrow: lateral sinus. Red arrow: torcular Herophili.

The venous blood of the brain is drained by three networks of cerebral veins: the superficial veins (cortical), the deep veins, and the veins of the posterior fossa. These veins terminate in the dural venous sinuses, which are themselves collected by the jugular veins [7]. The superior sagittal sinus, the straight sinus, and the occipital sinus converge toward the posterior confluence of the sinuses (torcular Herophili), located at the level of the internal occipital protuberance [8] [Figure 2]. From the torcular Herophili, venous blood flows into the jugular veins after travelling through the transverse and sigmoid sinuses. The dural sinuses contain the Pacchionian arachnoidian granulations that are used for the resorption of cerebrospinal fluid. The superficial system begins with the subcortical veins that drain the outer surfaces of the cortex; these subcortical veins drain into the pial veins located on the surface of the cortex. The pial veins drain into the cerebral veins, which are located on the surface of the brain.

**Figure 3:**
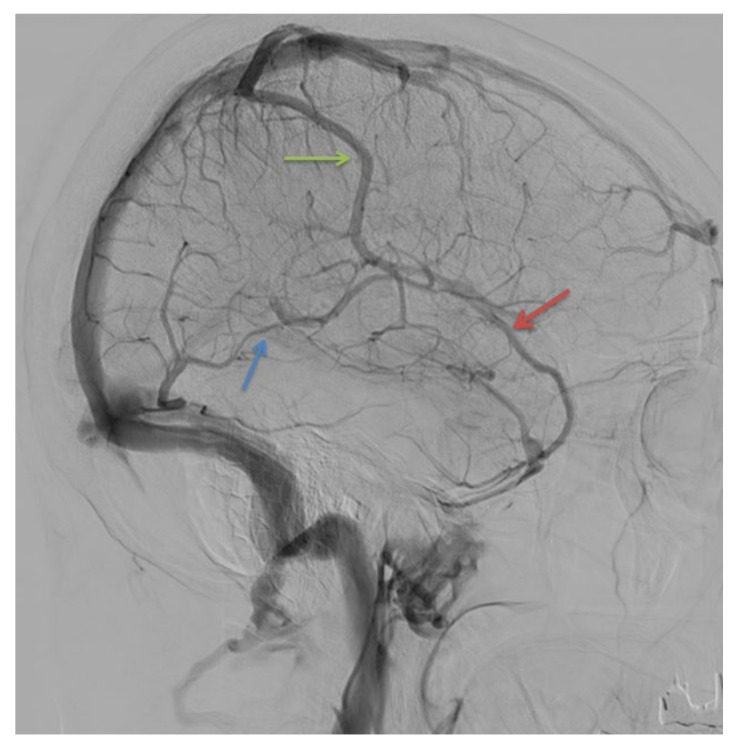
Digital subtraction angiography, sagittal plane. Red arrow: superficial middle cerebral vein. Green arrow: superior anastomotic vein (Trolard’s vein). Blue arrow: inferior anastomotic vein (Labbé’s vein).

There are many cerebral veins, but the three most important are the superficial middle cerebral veins; Trolard’s vein (also known as the superior anastomotic vein), which connects the superior sagittal sinus to the middle cerebral vein; and Labbé’s vein (also known as inferior anastomotic vein), which connects the middle cerebral vein to the lateral sinus [Figure 3].

The superficial cerebral veins can be divided into three collecting systems: a mediodorsal group draining into the superior sagittal sinus and straight sinus, a lateroventral group draining into the lateral sinus, and an anterior group draining into the cavernous sinus. These veins are connected by the superior anastomotic vein (Trolard’s vein), which connects the superior sagittal sinus to the middle cerebral veins. The inferior anastomotic vein (Labbé’s vein) connects the superior sagittal sinus and middle cerebral veins to the lateral sinus.

### MR Anatomy of the Superior Sagittal, Straight, Lateral and Torcular Sinuses

The superior sagittal single sinus drains most of the cortex. Its size increases from front to back. The anterior part may be absent and then replaced by two superior cerebral veins. It joins the straight sinus at the level of the torcular Herophili [Figure 2].

The inferior sagittal sinus, which is also single, drains the inner side of the middle part of the hemispheres and the corpus callosum.

**Figure 4:**
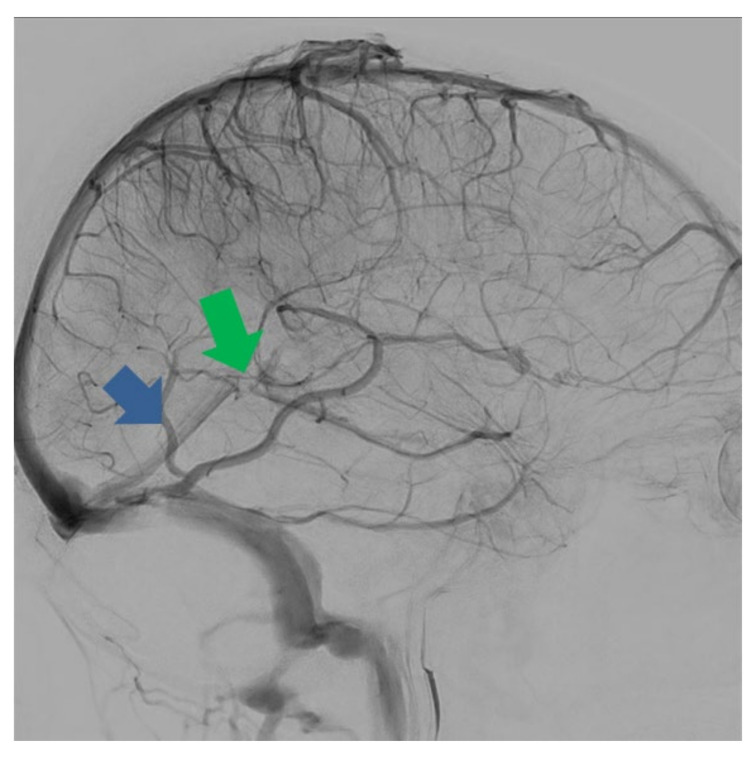
Digital subtraction angiography, sagittal plane. Anatomy of Galen’s ampulla and straight sinus. The straight sinus receives blood from the inferior sagittal sinus and the vein of Galen. It flows into the torcular Herophili, where it joins the superior sagittal sinus. Green arrow: Galen’s ampulla. Blue arrow: straight sinus.

The straight sinus, which is single, is the confluence of the vein of Galen and the inferior longitudinal sinus [Figure 4]. It drains into a transverse sinus (most often the left one) or into the torcular Herophili.

The lateral sinuses are divided into the transverse sinus and sigmoid sinus. They are usually asymmetrical, with the right lateral sinus frequently larger than the left. This asymmetry and the frequent hypoplasia of the left lateral sinus are important to be aware of so as to not interpret poor filling as a thrombosis of the left lateral sinus.

**Figure 5:**
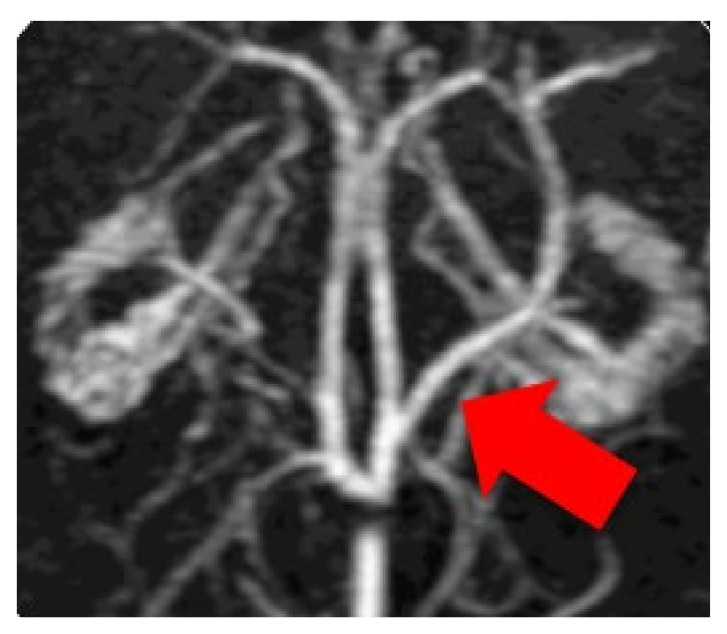
CE-MRV, axial and sagittal planes. MR anatomy of the basal veins. Red arrow: basal veins (former basal veins of Rosenthal).

**Figure 6:**
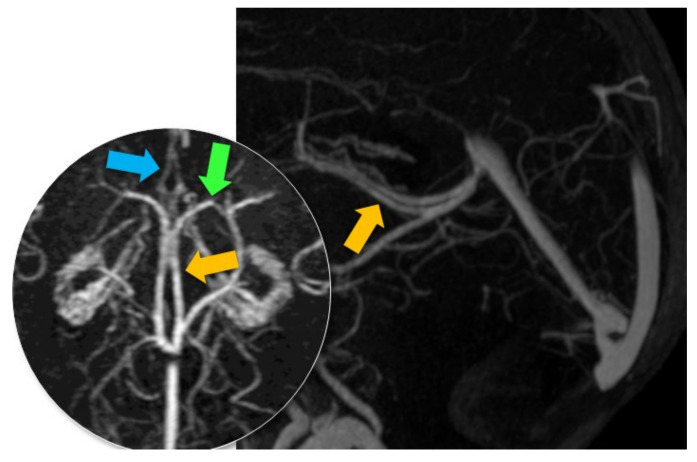
CE-MRV, axial and sagittal planes. MR anatomy of the septal, thalamostriate, and internal cerebral veins. Blue arrow: septal veins. Green arrow: thalamostriate veins. Yellow arrow: internal cerebral veins.

Thus, the torcular Herophili receives blood from the superficial veins through the superior sagittal sinus and blood from the deep veins through the straight sinus [Figure 5 and Figure 6].

**With symptoms suggesting cerebral venous thrombosis and knowledge of anatomy, what imaging techniques should be used?**

## 5. Brain Imaging

DSA has long been considered the gold standard for the diagnosis of CVT. Today, it is used almost exclusively to guide interventional radiology procedures. The diagnosis is based on CT, CT venography, MRI, and MR venography. DSA is still superior to MR venography and CT venography in terms of dynamic information and can yield important additional information, particularly in collateral venous drainage.

Noninvasive neuroimaging with CT and MRI allows for the rapid diagnosis of cerebral venous thrombosis and thus affects the prognosis by improving the speed of management and monitoring. It provides two types of findings: direct signs corresponding to the visualization of the thrombus or venous thrombosis and indirect signs such as the presence of cerebral ischemia or hemorrhage.

Direct signs include “positive” visualization of the thrombus on nonenhanced CT and MRI and “negative” visualization of the thrombotic material as a filling defect on CT venography and MR venography.

Indirect signs refer to brain changes in the brain parenchyma that occurred after the thrombosis and include venous edema, venous infarction, subarachnoid hemorrhage, and parenchymal hemorrhage. Unlike arterial infarcts, venous infarcts do not respect arterial territories or cross territory boundaries.

The different imaging techniques are mainly noninjected or postinjection CT with venous time acquisition and MRI with MR venography. CT provides less information on the clot, the vessels, and the parenchyma and few clues favoring intracranial hypertension [9]. CT venography was found accurate for diagnosing cerebral sinus thrombosis [10,11]. MRI provides information on the clot, the vessels, the parenchyma, and possible signs of intracranial hypertension [5,9,12,13].

Although less efficient than MRI for the detection of parenchymal abnormalities, CT is the first-line examination because of its greater availability [1]. It is used for examining patients with contraindications to MRI.

Both CT and MRI should look for local causes that may have contributed to the cerebral venous thrombosis, especially staphylococcal infection on the face, mastoiditis, or sphenoidal or ethmoidal sinusitis.

## 6. MRI

From a clinical point of view, in a patient presenting headache, the question that every radiologist asks is: Should I look for a ruptured brain aneurysm or cerebral venous thrombosis? If an aneurysm is clearly suspected, arterial 3D time of flight (TOF) imaging should be systematically performed, covering the posterior inferior cerebellar arteries to the callosal marginal arteries.

**Figure 7:**
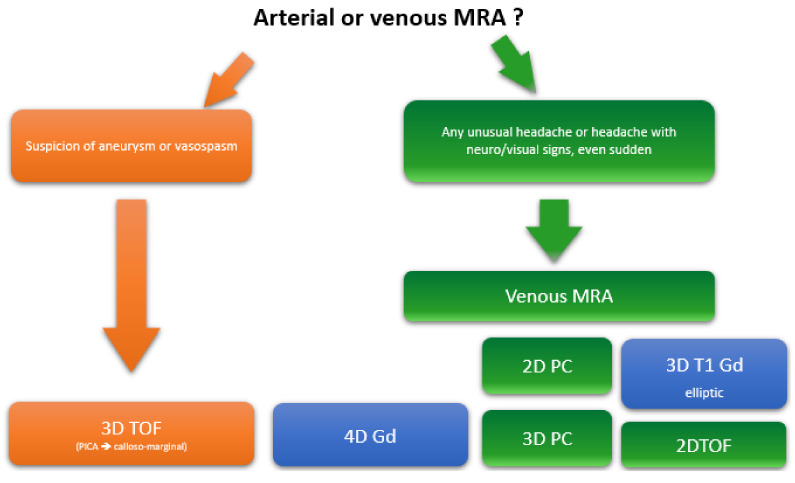
Decisional algorithm for choosing arterial or venous MR angiography. PC: phase contrast, Gd, gadolinium, TOF, time of flight.

In case of any unusual headache with neurological or visual signs or confusion, even if the onset is sudden, MR venography should be performed [4], with 2D or 3D phase contrast and 2D TOF or 3D T1 elliptical post-gadolinium enhancement [Figure 7].

Performed in the venous phase, 3D elliptical T1 post-gadolinium enhancement requires a bolus injection of gadolinium (0.1 mmol/kg with an injection flow rate of 2 to 3 mL/s) starting when the cerebral veins and/or the jugular veins are visible on the dynamic imaging detection of contrast arrival. Compared with 2D TOF and 2D phase contrast sequences, this sequence seems more efficient for visualizing the cortical veins, the deep system, and the sinuses of the base of the skull (petrous, cavernous, and basilar plexus). It also provides very good visualization of the lateral sinuses, even in cases of hypoplasia [12,14]. It also overcomes the limitations of other MR techniques, in particular signal losses in the event of slow or turbulent flows (2D TOF, 2D, and 3D phase contrast), not perpendicular to the acquisition plane (TOF) and in the event of inappropriate selection of encoding speed (2D and 3D phase contrast).

## 7. MRI Sequences and Semiology

**Figure 8:**
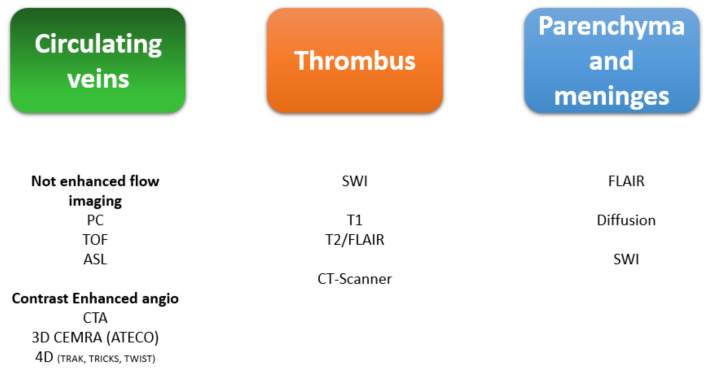
MRI sequences for imaging the veins, the thrombus, the parenchyma, and the meninges [15]. PC: phase contrast, TOF: time of flight, ASL: arterial spin labeling, CTA: CT angiography. 3D CE-MRA (ATECO): 3D contrast-enhanced MR angiography (auto-triggered elliptic centric ordered), 4D (TRAK, TRICKS, TWIST): dynamic contrast-enhanced MR angiography, FLAIR: fluid-attenuated inversion recovery, Diffusion: diffusion-weighted imaging, SWI: susceptibility-weighted imaging.

Distinctions should be made between vascular semiology, the thrombus signal, and any parenchymal or meningeal repercussions [Figure 8 and Table 1].

Parenchymal abnormalities are found in only 40% [8] of patients with cerebral venous thrombosis. They are edema and ischemic and/or hemorrhagic softening [4]. The edema presents a hyposignal on T1 sequences and a hypersignal on T2 sequences, whereas the hemorrhage is globally hypersignal on T1 and T2 sequences and hyposignal on T2 gradient-echo sequences. Ischemia and hemorrhage areas [4] cross arterial boundaries, readily bilateral and asymmetrical.

Thrombosis of the superior sagittal sinus is often accompanied by frontal or parietal lobar hematomas, whereas thrombosis of the lateral sinus is accompanied by temporal or occipital lesions. Deep parenchymal abnormalities involving the thalami suggest thrombosis of the vein of Galen or the straight sinus.

**Figure 9:**
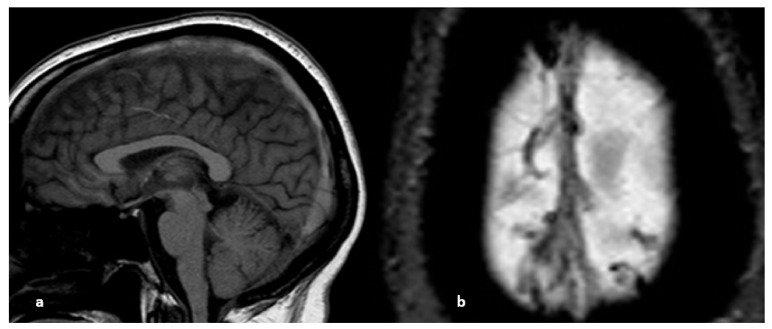
(**a**) Nonenhanced T1-weighted images, sagittal plane. Spontaneous hypersignal of the superior sagittal sinus suggests thrombosis. (**b**) T2* images, axial plane. Spontaneous hyposignal of the cortical veins of the vertex suggests cortical vein thrombi.

**Figure 10:**
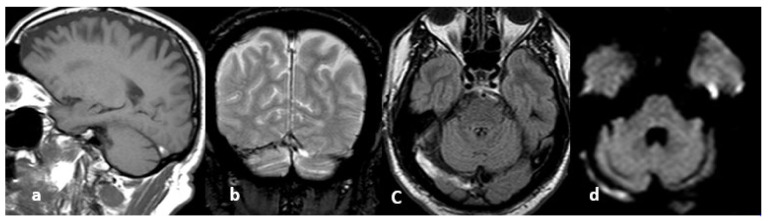
From left to right, spontaneous signal of the right lateral sinus suggesting thrombosis: (**a**) T1 hypersignal, (**b**) T2* hyposignal, (**c**) FLAIR hypersignal, (**d**) diffusion-weighted imaging hypersignal.

The thrombus signal evolves in the same way as a hematoma. The progressive changes in clot signal are related to the paramagnetic properties of hemoglobin. The acute thrombus generally presents a hyposignal on T2* sequences and susceptibility-weighted imaging (SWI) and a hypersignal on spin-echo T1, T2, diffusion-weighted imaging (DWI) and fluid-attenuated inversion recovery (FLAIR) sequences. SWI and T2 gradient-echo sequences are useful because they are sensitive to hemoglobin breakdown products that cause an exaggerated signal drop-out [Figure 9 and Figure 10]. However, the hyperintense signal on DWI has a lower sensitivity, but higher specificity, than the hyperintense on T2WI for the diagnosis of cerebral venous thrombosis [16,17].

**Figure 11:**
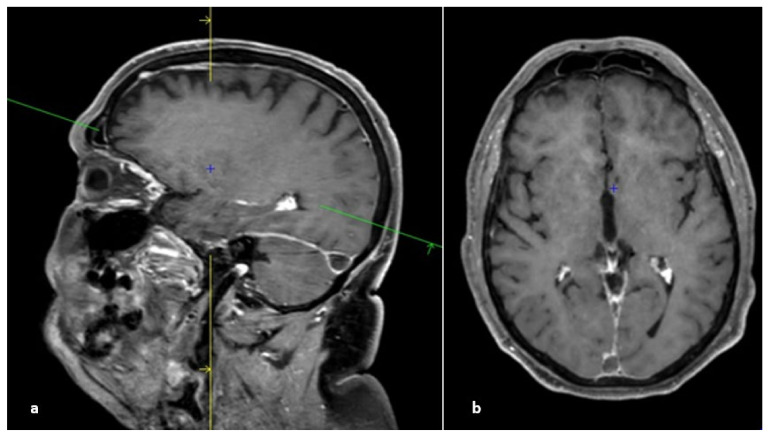
3D contrast-enhanced T1-weighted gradient-echo images. (**a**) Sagittal plane; “empty delta” sign in the lateral sinus. (**b**) Axial plane; empty delta sign in the superior sagittal sinus. The enhancement related to the injection occurs around the thrombus, which appears in relative hyposignal.

On enhanced morphological sequences after the injection of gadolinium, such as 3D T1 gadolinium enhancement, we look for a sign of “empty delta,” revealing venous enhancement around the thrombus [6,13], which is not particularly enhanced. To be positive, the empty delta sign must be visualized on several sections [Figure 11].

Beyond 15 days, the diagnosis of cerebral venous thrombosis can be delicate because of the possible partial recanalization.

**Figure 12:**
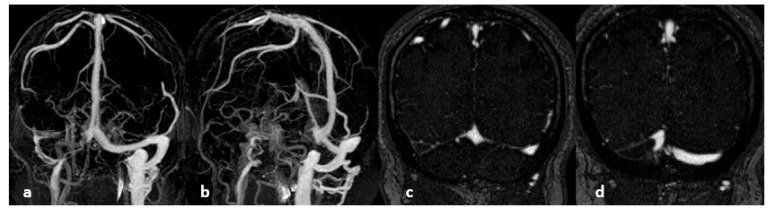
CE-MRV. From left to right: (**a**,**b**) maximum intensity projection (MIP) reconstructions; interruption of the proximal portion of the right lateral sinus; (**c**,**d**) native slices; defect in the lateral sinus suggests a thrombus.

On 3D contrast-enhanced MR venography (CE-MRV) auto-triggered elliptic centric ordered (ATECO), we can observe the filling defect of the vein [8] linked to the presence of the thrombus [Figure 12]. The ATECO technique allows the bolus to be synchronized with the acquisition.

Time-resolved MRA techniques: 4D-TRAK—Philips (4D Time Resolved Angiography using Keyhole), TRICKS—GE (Time Resolved Imaging of Contrast KineticS), TWIST—Siemens (time-resolved angiography with stochastic trajectories) allow for obtaining a sequential visualization of the arteries and then the veins and for answering the various questions when the symptomatology is too uncertain to engage directly in the search for cerebral venous thrombosis. A typical-time resolved MRA study might contain 20 images obtained at rates as rapid as 1–2 frames per second. An inherent trade-off exists between spatial and temporal resolution [18].

**Figure 13:**
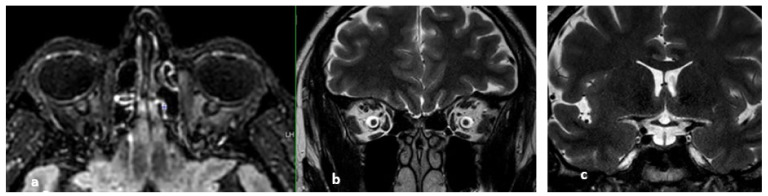
MRI signs of intracranial hypertension. They illustrate common findings in intracranial hypertension regardless of the presence of cerebral venous thrombosis. From left to right: (**a**) optic disc FLAIR hyperintensity, (**b**) dilation of the optic nerve sheaths, (**c**) empty sella turcica.

In case of intracranial hypertension, there will also be a dilation of the optic nerve sheaths and an empty sella turcica [Figure 13].

CE-MRV sequences should be differentiated from anatomical 3D T1 sequences such as SPGR, BRAVO, TFE, and FFE. These CE-MRV sequences are found in the manufacturers’ catalogs of injected vascular sequences; they are optimized to visualize the enhanced circulating structures [Table 2]. Therefore, the parenchyma has a weak signal. Because of the short echo time (TE) and the possible use of masks, the fat is also erased. These CE-MRV sequences most often remain interpretable when using half doses of gadolinium. As with CT, with CE-MRV, the delay between injection and acquisition is from 35 to 45 s, and the injection flow rate 1 to 3 cc/s.

MR venography sequences allow for an initial positive diagnosis of cerebral venous thrombosis and also for monitoring the thrombus and visualizing its partial or complete recanalization. Complete recanalization is not necessary for symptom improvement.

MR venography sequences can be performed without contrast injection such as TOF or phase contrast or after the intravenous injection of gadolinium [19]. However, injected techniques are superior to TOF and phase contrast [Table 3].

Injected MR venography offers the same type of information as CT venography by showing the filling defect with similar sensitivities and specificities.

The vascular filling sequences with gadolinium are more reliable because of no risk of false positives on slow flows.

Additionally, 3D contrast-enhanced MR venography allows for better characterization of the intracranial venous anatomy [19]. If there is a contraindication to the injection, phase contrast is preferred to 2D TOF [9]. However, these CE-MRV sequences present some easily circumvented interpretation pitfalls.

### What Is the Place of 3D Anatomical Sequences?

**Figure 14:**
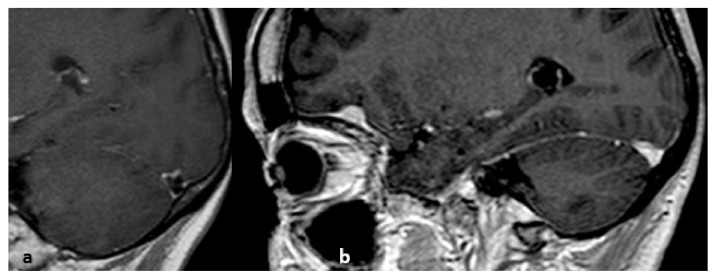
From left to right: (**a**) contrast-enhanced T1 spin echo, (**b**) contrast-enhanced T1 gradient echo.

If we acquire 3D anatomical T1 sequences after the injection of gadolinium-based contrast agents [9], we must remember that in gradient echo, the venous sinuses are filled with gadolinium, whereas in spin echo, the sinus is naturally empty [Figure 14]. Therefore, the semiology of the poor filling of the vessel can only be retained on the T1 gradient-echo sequences after the injection of gadolinium.

Therefore, T1 spin echo is not a good technique for assessing the presence of a thrombus in a dural sinus.

Of note, old thrombi can be enhanced if the sequences are acquired too long after the injection. In chronic thrombosis, these sequences can be misinterpreted because of the possible enhancement of the clot, which can then mimic a permeable sinus.

The signal of the thrombus also evolves as a function of time, as shown in Table 4 [21].

Along with vascular and parenchymal abnormalities, MRI signs of intracranial hypertension to look for include dilated optic nerve sheaths, papilledema, and empty sella turcica.

## 8. CT and Venous CT Scanning

### Technique and Results

As well as MRI, CT can highlight parenchymal and vascular abnormalities. Because of its wide availability, CT is used in emergency departments as a triage examination for neurological symptoms not suggesting arterial ischemia. CT exposes the patient to significant ionizing radiation [19]. However, results remain normal in 30% of cases [13]. On nonenhanced CT [22], we can visualize direct and indirect signs of cerebral venous thrombosis.

**Figure 15:**
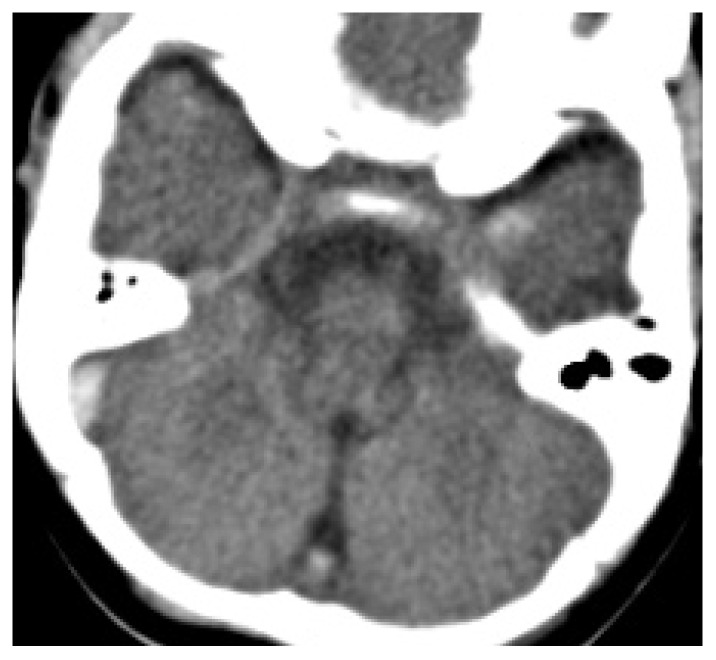
Nonenhanced CT. Spontaneous hyperdensity of the thrombus in the right lateral/sigmoid sinus junction.

Direct signs: a fresh thrombus spontaneously hyperdense in a dural sinus or a cortical vein usually called the “cord sign” [Figure 15] or dense clot sign [19,23,24,25]. The cord sign is best found in the first week of the disease, with estimated sensitivity of 64% and specificity of 97%. The spontaneous density of fresh blood gradually decreases after the first week. This sign is not very specific and can be falsely found in normal vessels with slow flow. Differential diagnoses are excessive visibility of circulating blood in venous structures in dehydrated patients, polycythemias, and increased hematocrit.

**Figure 16:**
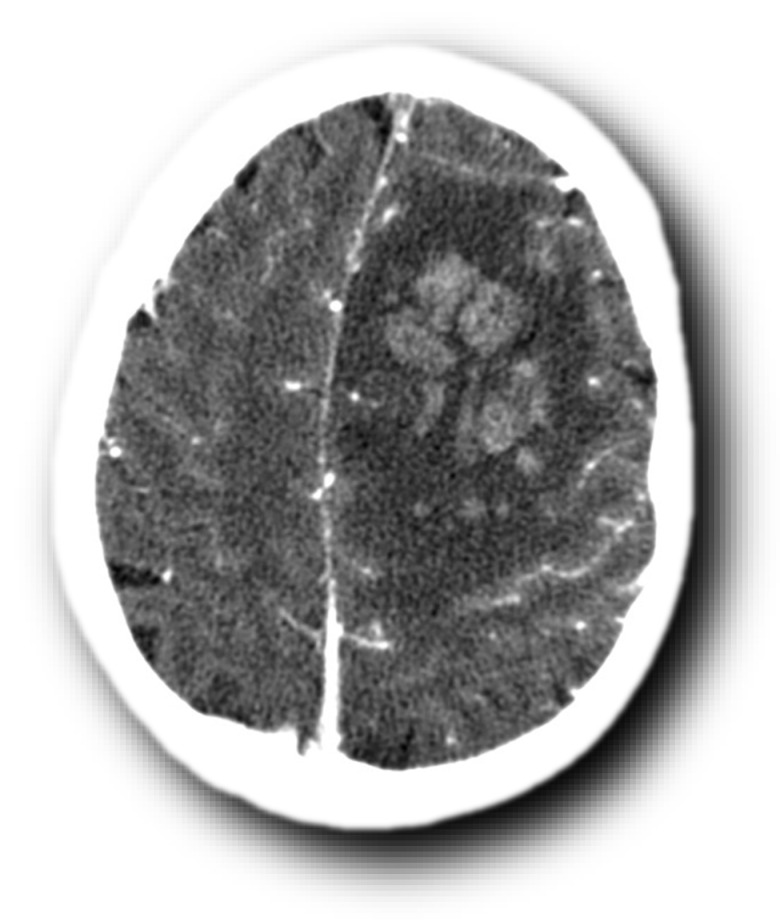
Left frontopolar venous hemorrhagic softening on CT.

Indirect signs: These occur in 60% to 80% of cases. The most common indirect signs evocative of cerebral venous thrombosis are multiple bilateral ischemic lesions or hematomas [Figure 16], bilateral thalamic edema, and temporo-occipital lesions [4,23]. Subarachnoid hemorrhage [10] or a herniation syndrome are also possibly seen.

The size of the ventricular cavities and good visibility of the sulci may be diminished in case of diffuse edema. Conversely, the volume of the ventricular cavities may sometimes be increased due to increased cerebrospinal fluid production and poor reabsorption. In these cases, the diagnosis of ventricular size change is greatly facilitated if the patient has previous neuroimaging findings.

The location of parenchymal abnormalities depends on the location of the thrombus and possible collateral supply routes. For example, deep abnormalities involving the internal capsule or thalami favor a deep venous thrombosis; temporo-occipital lesions suggest transverse sinus thrombosis and bilateral parasagittal hemispheric lesions of superior sagittal sinus thrombosis. The presence of a hemorrhagic focus or localized edema near a dural sinus favors a thrombosis of the sinus.

In case of suspected cerebral venous thrombosis on nonenhanced CT, the examination should be followed by CT venography or MR venography.

On iodinated contrast-enhanced CT, the injection is best performed with an automatic injector pushing 20 to 40 cc of iodinated contrast medium followed by physiological saline with an injection flow rate of 2 to 4 cc/sec. Volume acquisition starts from C1 to the vertex starting at 35 to 45 sec (or on the detection of arrival of the bolus in the right jugular vein) after the injection.

**Figure 17:**
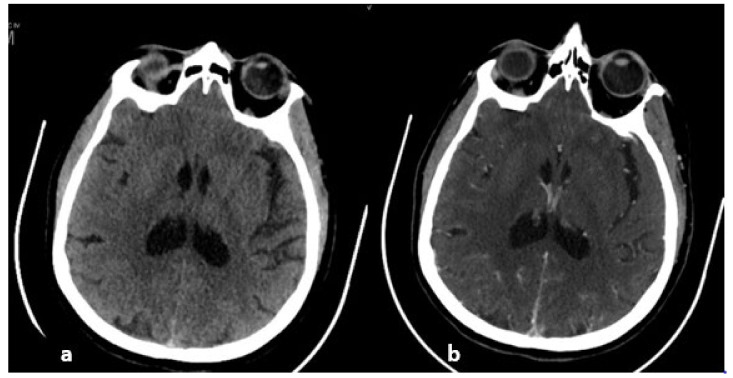
CT. From left to right: (**a**) nonenhanced CT: spontaneous hyperdensity of the superior sagittal sinus, (**b**) enhanced CT: empty delta sign testifying to the circulation of contrast around the thrombus, which appears as a defect of signal in the center of the superior sagittal sinus.

After intravenous injection [23], the most characteristic semiology is the empty delta sign [13], which results in a filling defect (corresponding to the thrombus) of contrast enhancement within a dural sinus enhanced in the periphery [6] [Figure 17]. To be positive, the sign of the empty delta must be visualized on several sections. The empty delta sign is most often visible between 5 days and 2 months after the event.

**Figure 18:**
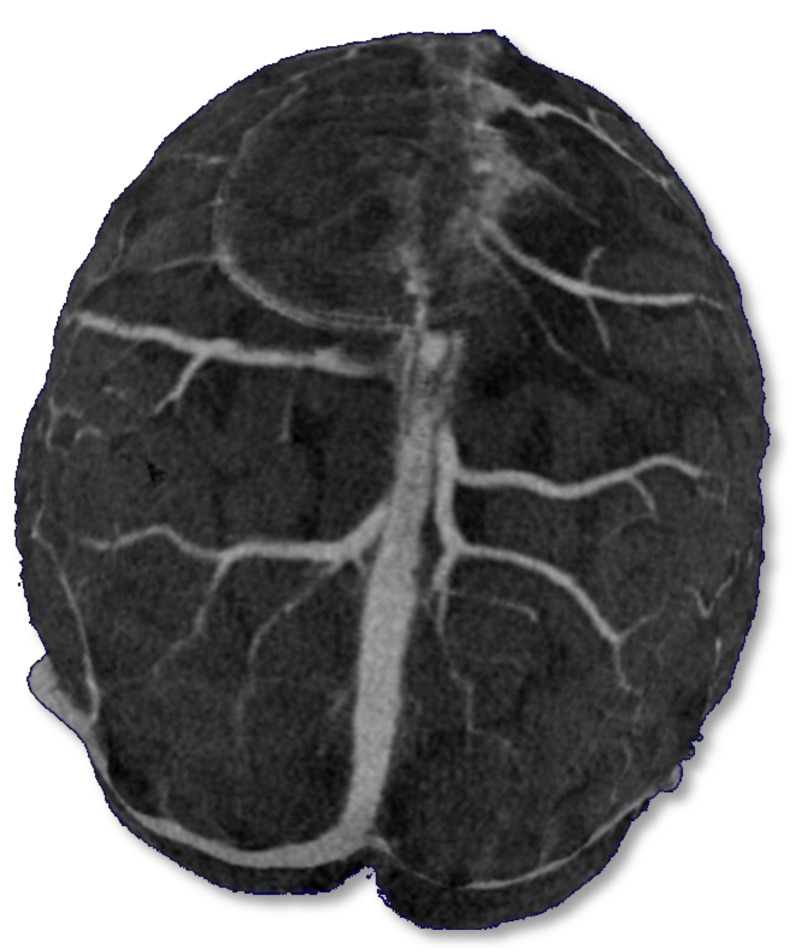
Volume rendering reconstructions of cerebral CT venography. Thrombosis of the anterior part of the superior sagittal sinus.

CT venography is less sensitive for the diagnosis of deep cortical venous thrombosis than for large dural sinus thrombosis; this can be improved by performing multiplanar reformations [Figure 18].

A false positive delta sign occurs when the superior sagittal sinus divides early. A split sinus or a septum within a sinus can also lead to a false positive diagnosis of cerebral venous thrombosis in an image with a pseudo empty delta sign. As in MRI, in CT, a chronic thrombus may enhance after iodinated contrast media injection and no longer give an empty delta sign.

The signs of intracranial hypertension, in particular the dilation of the optic nerve sheaths, are less well visualized than on MRI. The empty sella turcica remains clearly visible.

Again, normal CT results do not rule out cerebral venous thrombosis, and with any clinical doubt, complementary MRI should be performed.

## 9. Sensitivity and specificity

MRI: Several studies with evidence judged low compared MRI with MR venography with DSA acquisition. MR venography reliably demonstrated the large cerebral veins and sinus visualized on DSA. DSA was more sensitive than MR venography in evaluating the smaller cortical veins and the deep subcortical veins. In one study [26] of 20 patients with cerebral venous thrombosis, all documented by DSA, MRI with MR venography provided the diagnosis in all cases. When DSA was used as reference standard, CE-MRI had sensitivity and specificity 83% and 100%, respectively [27]. In another study of 92 patients [28], the sensitivity of MRI was 87%, specificity was 76.9%, positive predictive value was 94%, and negative predictive value was 58%.

The recommendation of the European Stroke Organization guidelines for the diagnosis of cerebral venous thrombosis is the use of MR venography as a reliable alternative to DSA for confirmation in patients with suspected cerebral venous thrombosis [5].

CT: Similarly, several studies with evidence judged low compared CT with CT venography with DSA. CT with CT venography was able to detect all cerebral venous thrombosis in patients with suspected cerebral venous thrombosis [29].

The recommendation of the European Stroke Organization guidelines for the diagnosis of cerebral venous thrombosis is the use of CT venography as a reliable alternative to DSA for the diagnosis of cerebral venous thrombosis [5].

In Xu’s meta-analysis, published in 2018 [30], 24 eligible articles comprising 48 studies (4595 cases) were included. The pooled sensitivity of the CT-CVT (cerebral venous thrombosis)/CT-CVST (cerebral venous sinus thrombosis) groups was 0.79 and the specificity was 0.90, respectively. For the MRI-CVT/MRI-CVST groups, the pooled sensitivity was 0.82 and the pooled specificity was 0.92, respectively.

### Digital Subtraction Angiography (DSA)

DSA for the positive diagnosis of cerebral venous thrombosis theoretically remains the most accurate method, but it has become exceptional because of noninvasive imaging. Cerebral angiography is performed only in cases of any doubt with CT venography or MR venography. The venous time must be visualized on at least two views. To improve diagnostic performance, the contralateral carotid artery is compressed. The diagnosis of cerebral venous thrombosis is easy with extensive thrombosis of the superior sagittal sinus, lateral sinus, or straight sinus [31]. The diagnosis is established on the typical presentation of a direct sign (absence of opacification of the thrombosed sinus) and indirect signs (presence of collateral circulation with venous dilatation and tortuous veins) but is more difficult when part of the sinus is not opacified or is irregular, in which case the indirect signs are more useful.

DSA is also used to guide exceptional therapeutic procedures such as endovascular mechanical thrombectomy used alone or combined with other techniques such as direct aspiration or pharmacological thrombolysis.

## 10. Summary of Imaging Support

If MRI is possible [13], 3D T1 turbo spin echo (TSE) without injection + 3D SWI + 3D FLAIR without injection + axial DWI, coronal T2 on the optical pathways; injection of gadolinium, then 3D CE-MR venography and 3D T1 gradient echo (for the veins) and spin echo if in doubt about an associated parenchymal or meningeal anomaly (e.g., meningioma), paying attention to the fact that the sinus is empty at the base in spin echo.

If MRI is impossible because not accessible or contraindicated, CT without injection can be followed by venous CT venography. However, CT exposes the patient to significant ionizing radiation [19].

## 11. Special Cases

### 11.1. Thrombosis of the Straight Sinus

**Figure 19:**
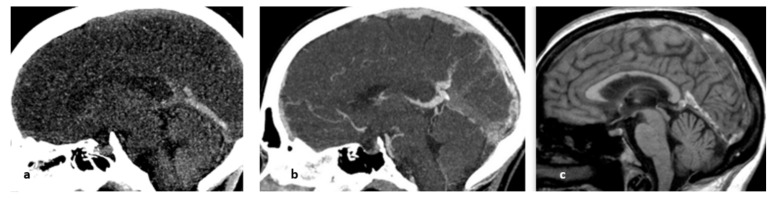
From left to right: (**a**) nonenhanced CT, sagittal plane; (spontaneous hyperdensity of the straight sinus); (**b**) after the injection of iodinated contrast media, absence of opacification of the straight sinus, thrombosis of the superior sagittal sinus above the torcular Herophili; (**c**) MRI, nonenhanced sagittal T1 sequence: spontaneous hypersignal of the thrombus.

**Figure 20:**
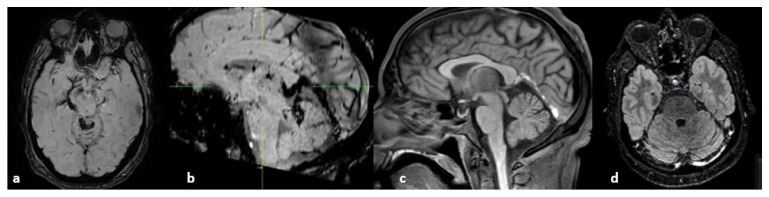
From left to right: (**a**,**b**) susceptibility-weighted imaging, axial (**a**) and sagittal (**b**) planes (thrombosis of the internal cerebral and basilar veins and straight sinus); (**c**) T1 sequence sagittal plane (spontaneous hypersignal of the thrombus in the straight sinus); (**d**) FLAIR image, axial plane (spontaneous hypersignal of the thrombus in the left lateral sinus).

**Figure 21:**
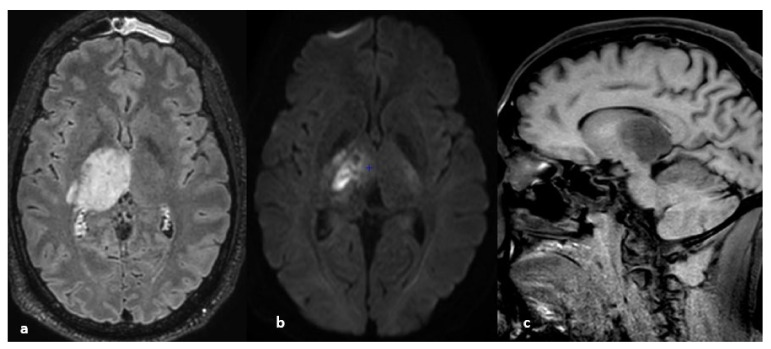

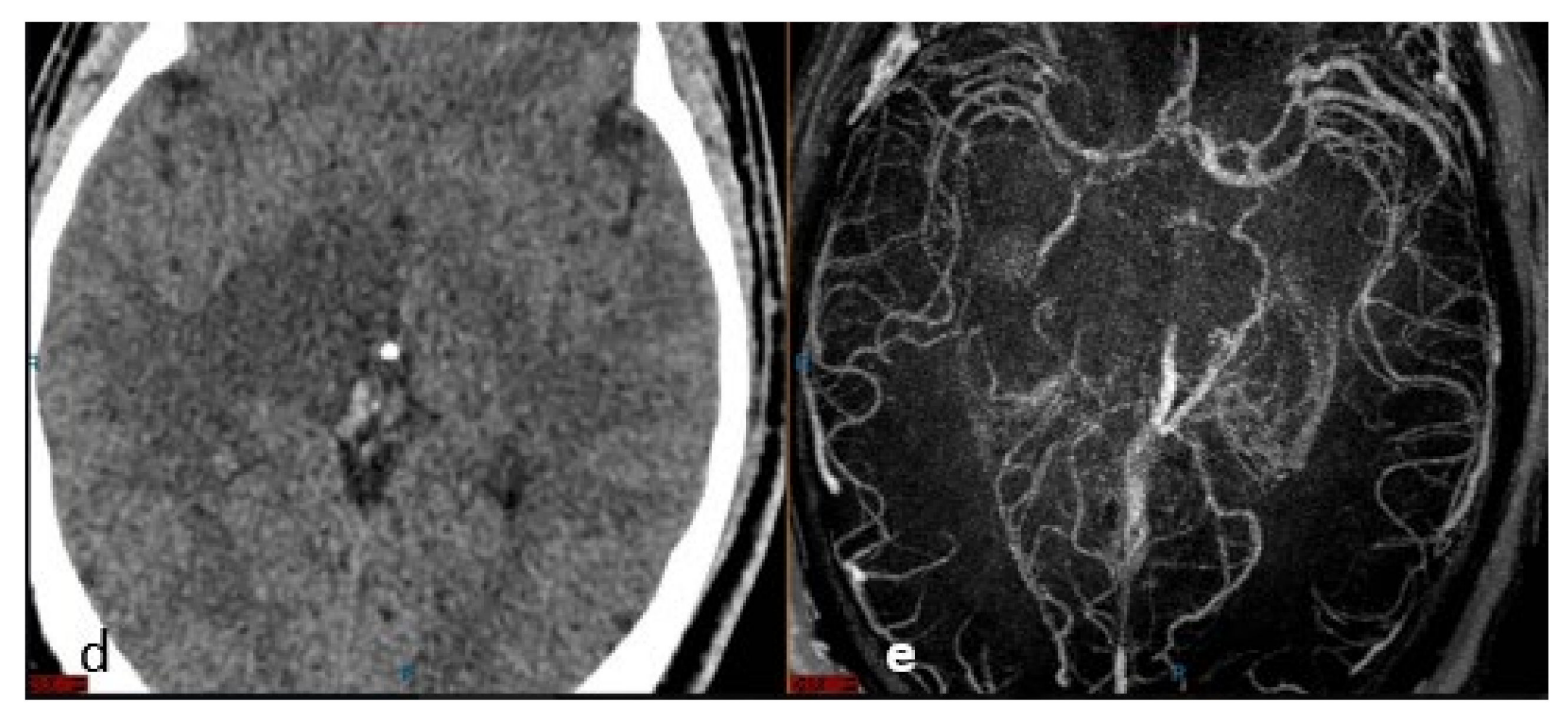
Ischemic lesion of the right thalamus. (**a**) FLAIR image, axial plane (spontaneous hypersignal of the right thalamus); (**b**) diffusion-weighted imaging; (hypersignal of the right thalamus); (**c**) nonenhanced T1 image, sagittal plane; (hyposignal of the right thalamus); (**d**) nonenhanced CT image (hypodensity of the right thalamus); (**e**) thrombosis of the right internal cerebral vein. Note the presence of left frontal sinusitis.

Straight sinus thrombosis is a classical but rare type of cerebral venous thrombosis. It is difficult to diagnose. It appears as deep ischemic changes with or without hemorrhagic parenchymal changes that are often thalamic or even bi-thalamic [1] [Figure 19, Figure 20 and Figure 21]. One of the differential diagnoses of straight sinus thrombosis is thrombosis of the artery of Percheron [31,32]. This artery is an anatomical variant. It is a single artery vascularizing the bi-thalamic territory. It usually arises from the first segment of one of the posterior cerebral arteries.

Bi-thalamic disorders classically present a clinical triad associating paralysis of verticality, memory disorders, and confusion. This triad manifests the same in arterial (Percheron’s artery) or venous (right sinus thrombosis) involvement.

### 11.2. Thrombosis of Labbé’s Vein

Labbé’s vein, also known as the inferior anastomotic vein, is part of the superficial venous system of the brain. It connects the superficial middle cerebral to the lateral sinus. CT and MR reveal parenchymal temporal lesions ipsilateral to the thrombosis and a spontaneously hyperdense (CT) and hypersignal T1 (MRI) extra-axial band corresponding to the thrombosed vein of Labbé [33].

### 11.3. Differential Diagnoses

**Figure 22:**
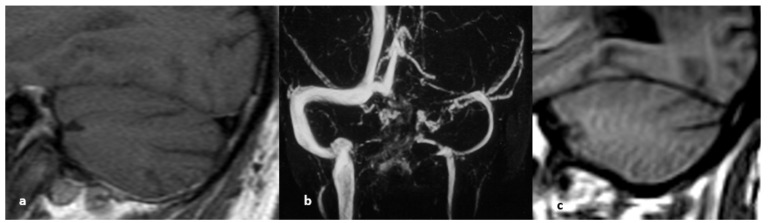
Left lateral sinus hypoplasia: (**a**) sagittal plane of the right lateral sinus; (**b**) phase contrast, lack of filling of the left lateral sinus; (**c**) sagittal plane of the right lateral sinus confirming its small caliber.

Hypoplasia of a lateral sinus: Attention must be paid to signal asymmetries linked to hypoplasia or aplasia of the lateral sinuses, particularly on the left side [Figure 22].

How to diagnose: With a hypoplastic lateral sinus, the groove of the lateral sinus, the jugular foramen, and the internal jugular vein are also hypoplastic on the same side, frequently with contralateral hypertrophy. In 3D FLAIR sequences, the asymmetry of the calibers of the two lateral sinuses can be visualized. The CE-MR venography sequences confirm the absence of a lacuna within the hypoplastic sinus. Finally, cases of frank hypoplasia frequently exhibit the presence of an occipital sinus, although it is not constant, especially with hypoplasia on the proximal third with a distal portion of the lateral sinus supplied by a large anastomotic vein.

**Figure 23:**
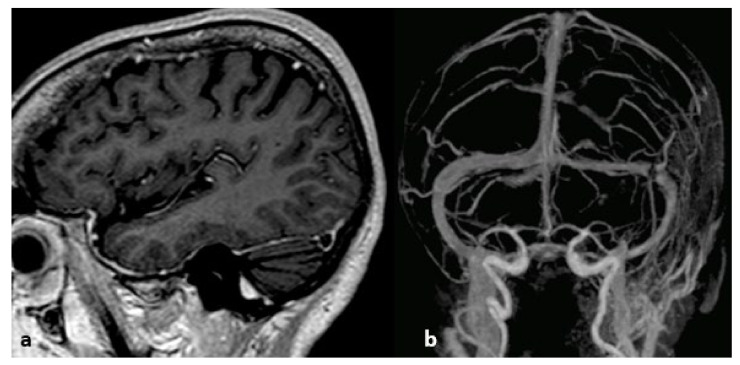
(**a**) Contrast-enhanced T1 image, sagittal plane (filling defect in the left lateral sinus); (**b**) CE-MRV confirms the typical round aspect of Pacchionian arachnoid granulations.

Pacchionian granulations [3,8]. Pacchionian arachnoid granulations are normal structures that protrude into the lumen of the dural sinus. When they are large, they can simulate a sinus thrombosis. They are most often seen in the transverse and superior sagittal sinuses. With the improvement in MRI spatial and contrast resolution, filling defects corresponding to arachnoid granulations are increasingly better visualized [Figure 23].

Arachnoid granulations must be differentiated from filling defects secondary to sinus thrombosis. Arachnoid granulations typically have a signal like that of cerebrospinal fluid and appear as focal filling defects with a characteristic anatomical distribution. They are often rounded and adherent to the wall of the dural sinus. After injection, they may present heterogeneous central enhancement.

Termination of the superior sagittal sinus above the torcular in the right lateral sinus. A high or asymmetric bifurcation may resemble an intra-sinus thrombus and induce a false empty delta sign [21]. The assessment of sequential images and the documentation of venous continuity are necessary to avoid this pitfall.

Conclusion: MRI and/or cerebral CT are reliable alternatives to DSA for imaging cerebral venous thrombosis. They are essential for the positive diagnosis of cerebral venous thrombosis. CT venography and MR venography are equally accurate for diagnosing cerebral venous thrombosis. The advantage of CT is the rapid acquisition, and the disadvantage is the significant exposure to ionizing radiation. MRI has the advantage of showing the thrombus itself and being more sensitive in detecting parenchymal lesions. Techniques may need to be combined to avoid pitfalls. The semiology of the nonenhanced CT should not be ignored because this examination remains widely practiced in cases of nonspecific neurological symptoms. Contrast-enhanced MRI is more accurate than non–contrast-enhanced MRI for diagnosing cerebral venous thrombosis and CT venography is more accurate than CT alone [23]. The radiologist must master the cerebral venous sinus anatomy and the different acquisitions to best orient the examination and avoid the pitfalls.

## Figures and Tables

**Table 1:** MRI results according to sequences.

	Thrombus-Veins	Parenchyma	Optic Nerves	Sella
TI	Loss of flow hyposignal Thrombus in iso/hypersignal	Hypo/isosignaledema Hemorrhages-Petechiae		
T2	Thrombus in hypo/iso signal at the acute stage then hypersignal	High signal edema Sometimes: Small ventricles and ptosis of the cerebellar tonsils. Parenchymal hematoma	Intracranial hypertension: dilation of the optic nerve sheaths. Papilledema	intracranial hypertension: “Empty” sella turcica.
Axial T2* or SWI	Frank hyposignal. High sensitivity for cortical veins.	Hemorrhages-Petechiae.		
Axial DWI.	Thrombus in hypersignal	Hypersignal B1000 and decreased ADC in ischemic lesions.		
Enhanced 3DT1EG	Intraluminal defect			
Non-enhanced Venous MRA	Specifies the location of the occlusion.			
CE-MRA	Defect in the vein. Not very sensitive to slow flows. Good filling of the cortical veins.			
3D Flair	Thrombus in hypersignal.	Edema. Intra parenchymal hematoma. Subarachnoid hemorrhage.		

SWI: susceptibility-weighted imaging. DWI: diffusion-weighted imaging, CE-MRA: contrast-enhanced magnetic resonance angiography, ADC: apparent diffusion coefficient, FLAIR: fluid-attenuated inversion recovery.

**Table 2:** Pitfalls of contrast-enhanced elliptical 3D sequences [8].

Risk	Solution
Acquisition too early: part of the venous system is not opacified	Immediately launch a new acquisition
	Using anatomical sequences: thrombus signal
	Complete with CT angiography
Missing a partial thrombosis on the MIP	Watch native images
Missing an old thrombosis (enhancement of the thrombus)	Do not use long and/or delayed injected sequences
Confusing Pacchionian granulation with thrombosis	Semiological knowledge

**Table 3:** Advantages and disadvantages of techniques [8,20]. Gado: gadolinium, TOF: time of flight, PC: phase contrast. Elliptic 3D T1: CE-MRV.

Sequence	Gado	Duration	Advantages	Disadvantages
2D TOF	No	4 min	No contrast injection Arteries removed	Slow flow Cortical veins Spatial resolution
3D PC	No	6 min	No contrast injection Less sensitive to slow flows than 2DTOF	Slow flows Long Acquisition Spatial resolution
2D PC	No	2 min	No contrast injection Fast Acquisition No reconstructions	Slow flows Cortical vein Spatial resolution
Elliptic-centric 3D T1	Yes	1 min	Insensitive to slow flows (no false positives) Cortical veins	Not feasible if injection contraindicated Arteries not removed May contain fat interposition

**Table 4:** The evolution of the thrombus signal.

	Normal Sinus	Thrombus Less than 5 Days Old	Thrombus from D5 to D30	Thrombus Older than 1 Month
T1	Hyposignal	Isosignal	Hypersignal	Iso/Hypersignal
T2	Hyposignal	Hypo/Isosignal	Iso/Hypersignal	Iso/Hypersignal
Contrast-enhanced T1	Homogeneous because the fresh thrombus can be enhanced	Empty Delta sign	Empty Delta sign	Empty Delta
